# The rs1051931 G>A Polymorphism in the *PLA2G7* Gene Confers Resistance to Immunoglobulin Therapy in Kawasaki Disease in a Southern Chinese Population

**DOI:** 10.3389/fped.2020.00338

**Published:** 2020-06-23

**Authors:** Xueping Gu, Wenchun Lin, Yufen Xu, Di Che, Yaqian Tan, Zhaoliang Lu, Lei Pi, Lanyan Fu, Huazhong Zhou, Zhiyong Jiang, Xiaoqiong Gu

**Affiliations:** ^1^Department of Blood Transfusion and Clinical Lab, Guangzhou Women and Children's Medical Center, Guangzhou Institute of Pediatrics, Guangzhou Medical University, Guangzhou, China; ^2^Department of Pneumology, Guangzhou Women and Children's Medical Center, Guangzhou Medical College, Guangzhou, China; ^3^Department of Clinical Biological Resource Bank, Guangzhou Women and Children's Medical Center, Guangzhou Institute of Pediatrics, Guangzhou Medical University, Guangzhou, China

**Keywords:** rs1051931, polymorphism, PLA2G7, immunoglobulin therapy, Kawasaki disease

## Abstract

**Background:** Kawasaki disease (KD) is a common cardiovascular disease in infants and young children, with fever, rash, and conjunctivitis as the main clinical manifestations, which can lead to the occurrence of coronary aneurysms. Intravenous immunoglobulin (IVIG) is the preferred treatment for KD patients, but 10–20% of patients are resistant to IVIG. Lipoprotein-associated phospholipase A 2 (Lp-PLA2) is a potential therapeutic target for coronary atherosclerotic heart disease, and the polymorphism of Phospholipase A2 Group VII (*PLA2G7*) is closely related to the activity of Lp-PLA2, of which rs1051931 is the strongest. Therefore, the rs1051931 polymorphism may be a predictor of IVIG resistance in KD patients.

**Methods:** A total of 760 KD cases, including 148 IVIG-resistant patients and 612 IVIG-responsive patients, were genotyped for rs1051931 in *PLA2G7*, we compared the effects of rs1051931 on IVIG treatment in KD patients by odds ratios (OR) and 95% confidence interval (CI).

**Results:** The homozygous mutation AA may be a protective factor for IVIG resistance in KD patients (adjusted OR = 3.47, 95% CI = 1.14–10.57, *P* = 0.0284) and is more evident in patients with KD aged <60 months (adjusted OR = 3.68, 95% CI = 1.10–12.28, *P* = 0.0399).

**Conclusions:** The *PLA2G7* rs1051931 G>A polymorphism may be suitable as a biomarker for the diagnosis or prognosis of IVIG resistance in KD in a southern Chinese population.

## Introduction

Kawasaki disease (KD), known also as Kawasaki syndrome or mucocutaneous lymph node syndrome, is an acute systemic vasculitis that primarily affects infants and young children (1, 2). The disease was first described in Japan by Dr. Tomisaku Kawasaki in 1967 and is now recognized as the leading cause of acquired heart disease among children in industrialized countries, especially in Asian countries (3, 4). The cardiac complications of KD are coronary artery aneurysms, coronary artery dilatation, and even myocardial infarction (5). The pathogenesis of KD is still unclear, but the immune response, microbial infections, and genetic factors are considered to contribute to its development. Approximately 25% of untreated KD patients developed coronary artery complications (6). The standard treatment for KD is intravenous immunoglobulin (IVIG), which can reduce both fever duration and the incidence of coronary artery lesions (CAL). However, despite receiving IVIG treatment, fever persists in 10–15% of the patients (7), and patients with IVIG resistance have a higher risk of CAL (8). Thus, predicting IVIG resistance in patients with KD is very important.

*PLA2G7* encodes plasma PAF acetylhydrolase (PAF-AH), an extracellular Lp-PLA2, whose activity is related to large-artery atherosclerotic etiology and recurrent stroke in transient ischaemic attack patients (9). The most interesting SNP of *PLA2G7* is Ala379Val (rs1051931), which has been associated with circulating Lp-PLA2 and atherosclerotic disease (10, 11). The association of the rs1051931 G>A genetic polymorphism with IVIG insensitivity in patients with KD is unknown so far. In this study, we focused on whether the *PLA2G7* rs1051931 G>A polymorphism was related to resistance to IVIG therapy in KD patients.

## Materials and Methods

### Study Subjects

We collected 148 IVIG-resistant patients and 612 IVIG-responsive patients with KD. The patients were derived from a portion of the KD cases collected from January 2012 to January 2017 at the Guangzhou Women and Children's Medical Center in Guangzhou, China. KD patients were diagnosed based on the American Heart Association's KD diagnostic criteria in 2004 (8). IVIG resistance is determined by persistent or recrudescent fever at least 36 h after completion of the first IVIG infusion (8). The KD patients, as outpatients with follow-ups and as inpatients, attended our hospital. This study was approved by the Guangzhou Women and Children's Medical Center Ethics Committee (2014073009), and with informed consent of the children and their families.

### SNP Genotyping

Genomic DNA was extracted from anticoagulant-containing peripheral blood collected from patients using the TIANamp Blood DNA Kit (DP318, TIANGEN Biotech, Beijing) according to the manufacturer's instructions. The procedures can be found in our previous paper (12). The *PLA2G7* rs1051931 G>A polymorphism was genotyped with TaqMan method. Allele-specific probes were ordered from Applied Biosystems. PCR was performed in 384-well plates with an ABI-Q6 Sequence Detection System machine (Thermo Fisher Scientific). Additionally, in order to ensure the quality and accuracy of the genotyping results, we randomly selected 10% of the samples for repeated analysis, and the results were completely consistent.

### Statistical Analysis

We first examined the Hardy-Weinberg equilibrium (HWE) of the samples. Next, we use χ^2^ test to evaluate the significant differences between IVIG-resistant cases and IVIG-responsive cases in the frequency distributions and genotypes. Odds ratios (OR) and 95% confidence intervals (CI) were used to quantify the association between the *PLA2G7* rs1051931 G>A polymorphism and the susceptibility of IVIG treatment in KD patients with adjustments for age and gender. The association between the *PLA2G7* rs1051931 G>A polymorphism and resistance to IVIG treatment in KD cases was evaluated by age and gender stratification analysis. Statistical analyses were performed by SAS software (Version 9.4; SAS Institute, Cary, NC, USA).

## Results

### Demographic Characteristics

**Figure 1:**
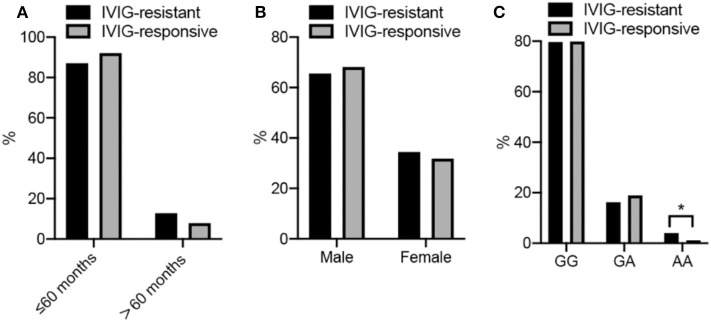
**(A–C)** Show the frequency disturbion of age, sex, and genotype in KD cases, respectly. More detailed data are described in the Tables.

A total of 148 IVIG-resistant cases and 612 IVIG-responsive cases were analyzed in this study. The demographics of participants are all shown in Figures 1A,B and Table 1. The mean ages were 29 months (29 ± 25, range from 1 to 125 months) for IVIG-resistant patients and 28 months (28 ± 23, range from 1 to 156 months) for IVIG-responsive patients. There showed no significant differences in age (*P* = 0.656) or gender (*P* = 0.5462) between the IVIG-resistant and IVIG-responsive KD patients.

**Table 1:** Frequency distribution of selected characteristics in KD cases.

**Variables**	**IVIG-insensitive^a^, No. (%)**	**IVIG-sensitive^b^**	***P^c^***
All subjects	148 (100)	612 (100)	
Age range, months	1.00–125.00	1.00–156.00	
Mean ± SD, months	29.02 ± 25.37	27.90 ± 23.11	
≤60 months	129 (87.16)	564 (92.16)	0.0656
>60 months	19 (12.84)	48 (7.84)	
Sex			
Male	97 (65.54)	417 (68.14)	0.5462
Female	51 (34.46)	195 (31.86)	

a *Kawasaki disease patients who were insensitive to IVIG therapy*.

b *Kawasaki disease patients who were sensitive to IVIG therapy*.

c *Two-sided χ^2^-test' for distributions between Kawasaki disease patients with IVIG insensitivity and IVIG sensitivity*.

### Association Between the *PLA2G7* rs1051931 G>A and the Resistance to IVIG in KD Patients

The genotype distributions of the *PLA2G7* rs1051931 G>A polymorphism in the IVIG-resistant and IVIG-responsive KD patients are shown in Figure 1C and Table 2. The genotype frequency distributions of the *PLA2G7* rs1051931 polymorphisms were 79.73% (GG), 16.22% (GA), and 4.05% (AA) in the IVIG-resistant group and 79.90% (GG), 18.95% (GA), and 1.14% (AA) in the IVIG-responsive group. In comparison with IVIG-resistant and IVIG-responsive KD subjects, there were significant differences in the AA genotype of *PLA2G7* rs1051931 (AA vs. GG: adjusted OR = 3.47, 95% CI = 1.14–10.57, *P* = 0.0284; AA vs. GG+GA: adjusted OR = 3.57, 95% CI = 1.18–10.84, *P* = 0.0247), which means KD patients with AA mutation were more resistant to IVIG (*P* = 0.0281).

**Table 2:** Genotype frequency distribution of *PLA2G7* rs1051931 in KD cases.

**Genotype/alleles** **rs1051931 G/A**	**IVIG-insensitive, No. (%)**	**IVIG-sensitive, No. (%)**	***P*^a^**	**Crude OR (95% CI)**	***P***	**Adjusted OR^b^ (95% CI)^b^**	***P*^b^**
GG	118 (79.73)	489 (79.90)	0.0735	1.00	/	1.00	/
GA	24 (16.22)	116 (18.95)		0.86 (0.53–1.39)	0.5327	0.86 (0.53–1.40)	0.5437
AA	6 (4.05)	7 (1.14)		3.55 (1.17–10.77)	**0.0251**	3.47 (1.14–10.57)	**0.0284**
GA/AA	30 (20.27)	123 (20.10)	0.9626	1.01 (0.65–1.58)	0.9625	1.01 (0.65–1.58)	0.9604
GG/GA	142 (95.95)	605 (98.86)	**0.0281**	1.00	/	1.00	/
AA	6 (4.05)	7 (1.14)		3.65 (1.21–11.03)	**0.0217**	3.57 (1.18–10.84)	**0.0247**
G	260 (87.84)	1094 (89.38)	0.4510	1.00	/	1.00	/
A	36 (12.16)	130 (10.62)		1.17 (0.79–1.73)	0.4459	1.16 (0.79–1.73)	0.4518

a *Two-sided χ^2^-test' for distributions between Kawasaki disease patients who were insensitive to IVIG treatment and who were sensitive to IVIG treatment*.

b *Adjusted for age and gender status in logistic regress models. The bold values indicate Statistical differences*.

### Stratification Analysis

We further explored the association of the *PLA2G7* rs1051931 polymorphism with IVIG resistance in KD in the stratified analysis by age and gender. For KD strikes predominantly children younger than 5 years of age (2), so we analyzed the rs1051931 GG/GA variant in children ≤60 months, the result showed that AA genotype may be more protective (adjusted OR = 3.68, 95% CI = 1.10–12.28, *P* = 0.0399) (Table 3). However, there showed no significant associations with other stratified analyses.

**Table 3:** Stratification analysis of susceptibility in Kawasaki disease patients.

**Variables**	**rs1051931 (IVIG-sensitive/IVIG-insensitive)**		***P***	**Crude OR**	***P***	**Adjusted OR^a^**	***P*^a^**
	**GG/GA**	**AA**					
Age, months							
≤60	124/558	5/6	**0.0402**	3.75 (1.23–12.48)	**0.0312**	3.68 (1.10–12.28)	**0.0399**
>60	18/47	1/1	0.5124	2.61 (0.16–43.99)	0.5056	2.42 (0.14–41.86)	0.5445
Gender							
Male	94/412	3/5	0.2146	2.63 (0.62–11.20)	0.1909	2.27 (0.52–9.86)	0.2728
Female	48/193	3/2	0.0532	6.03 (0.98–37.08)	0.0526	5.91 (0.96–36.58)	0.0561

a *Adjusted for age and gender. The bold values indicate Statistical differences*.

## Discussion

KD is the most common cause of acquired heart disease in infants and children, especially in developed countries. High-dose IVIG treatment significantly reduced the risk of CAL, but some children with KD failed to respond to initial IVIG therapy. Therefore, early identification of the risk factors of IVIG resistance is important. Our research showed that the *PLA2G7* rs1051931 AA genotype was associated with a protective effect against IVIG resistance for KD and that the effect was more evident in children ≤60 months of age. To the best of my knowledge, this is the first study in which *PLA2G7* rs1051931 G>A polymorphisms were found to be related to IVIG response in KD patients.

The mechanism of action of IVIG in KD is unclear. Potential explanations include immunologic blockade of the Fc receptor (13), interaction with dendritic cells (14)/T cells (15) and NK cells (16), an increase in the production of antibodies against the specific aetiologic agent (17), or downregulation of cytokine production (18). It is certainly plausible that IVIG interacts with many arms of the immune and vascular systems to achieve the downregulation of inflammation. The main purpose of IVIG treatment of KD is to guard against coronary artery damage and to reduce levels of tissue inflammation.

Experimental studies have shown that *PLA2G7* may be an effective therapeutic target in humans and animals, assuming that this enzyme is related to oxidative modification of LDL and the development of arterial inflammation (19). *PLA2G7* rs1051931 was nominally associated with Lp-PLA2 activity (20). The substitution of Ala by Val in A379V leads to twofold decrease in the affinity of Lp-PLA2 for its substrate, and thus reduces the degradation of PAF (21). The reduced activity of Lp-PLA2 due to potential genetic propensity may be an important factor that predisposes an individual toward IVIG resistance or involved in the pathogenesis of KD. *PLA2G7* rs1051931 polymorphisms were found to be associated with coronary artery disease (11, 22). The SNP rs1051931 was not associated with any of the cardiovascular risk factors (23–26). However, the rs1051931 variant in coronary artery disease patients is associated with a high risk of myocardial infarction (27). A previous study showed that the *PLA2G7* V279F polymorphism (G/T transversion) and consequent enzymatic deficiency is one of the factor for IVIG resistance in Japanese patients with acute KD (28), which is consistent with our results that mutant allele of *PLA2G7* may involve in IVIG therapy for KD patients. According to these studies, PAF-AH could be important in protecting tissues by degrading PAF and oxidized phospholipids into biologically inactive molecules.

Although this is the first investigation of the association between the *PLA2G7* rs1051931 G>A polymorphism and IVIG resistance in southern Chinese KD patients, our study has potential limitations that should be reviewed. First of all, this study was limited to a southern Chinese population, and cases from other ethnic groups were not assessed. Second, we examined only the rs1051931 G>A polymorphism; other potential SNPs of *PLA2G7* were not included. Third, the number of IVIG resistant patients is insufficient, and further studies with larger sample size are needed to confirm our results.

Overall, we conclude that the A379V polymorphism in the *PLA2G7* gene may be a potential factor for IVIG resistance in southern Chinese patients with KD, and it could be suitable as a biomarker for the diagnosis or prognosis of IVIG resistance in KD.

## Data Availability Statement

The raw data supporting the conclusions of this article will be made available by the authors, without undue reservation, to any qualified researcher.

## Ethics Statement

Written informed consent was obtained from the individual(s), and minor(s)' legal guardian/next of kin, for the publication of any potentially identifiable images or data included in this article.

## Author Contributions

All authors contributed significantly to this work. XuG, ZJ, and XiG were primarily responsible for the overall project design and paper writing. WL and YX were responsible for performing the experiments. Data analysis was carried out by YX and DC. DC and XiG modified and polished the manuscript. The others give suggestion for this article. All authors have reviewed and approved the manuscript.

## Conflict of Interest

The authors declare that the research was conducted in the absence of any commercial or financial relationships that could be construed as a potential conflict of interest.
